# Effects of Mefloquine Use on *Plasmodium vivax* Multidrug Resistance

**DOI:** 10.3201/eid2010.140411

**Published:** 2014-10

**Authors:** Nimol Khim, Voahangy Andrianaranjaka, Jean Popovici, Saorin Kim, Arsene Ratsimbasoa, Christophe Benedet, Celine Barnadas, Remy Durand, Marc Thellier, Eric Legrand, Lise Musset, Michela Menegon, Carlo Severini, Bakri Y.M. Nour, Magali Tichit, Christiane Bouchier, Odile Mercereau-Puijalon, Didier Ménard

**Affiliations:** Institut Pasteur, Phnom Penh, Cambodia (N. Khim, J. Popovici, S. Kim, C. Benedet, D. Ménard);; Institut Pasteur, Antananarivo, Madagascar (V. Andrianaranjaka);; Université d’Antananarivo, Antananarivo (A. Ratsimbasoa);; Walter and Eliza Hall Institute of Medical Research, Melbourne, Victoria, Australia (C. Barnadas);; University of Melbourne, Melbourne (C. Barnadas);; Hôpital Avicenne, Bobigny, France (R. Durand);; Centre Hospitalier Universitaire Pitié Salpetrière, Paris, France (M. Thellier);; Institut Pasteur, Cayenne, French Guiana (E. Legrand, L. Musset);; Istituto Superiore di Sanità (ISS), Rome, Italy (M. Menegon, C. Severini);; University of Gezira, Wad Medani, Sudan (B.Y.M. Nour);; Institut Pasteur, Paris (M. Tichit, C. Bouchier, O. Mercereau-Puijalon)

**Keywords:** Plasmodium vivax, drug resistance, mdr-1 gene, mefloquine, copy number, malaria, parasites, Africa, Asia, South America

## Abstract

Numerous studies have indicated a strong association between amplification of the *multidrug resistance-1* gene and in vivo and in vitro mefloquine resistance of *Plasmodium falciparum*. Although falciparum infection usually is not treated with mefloquine, incorrect diagnosis, high frequency of undetected mixed infections, or relapses of *P. vivax* infection triggered by *P. falciparum* infections expose non–*P. falciparum* parasites to mefloquine. To assess the consequences of such unintentional treatments on *P. vivax*, we studied variations in number of *Pvmdr-1* (PlasmoDB accession no. PVX_080100, NCBI reference sequence NC_009915.1) copies worldwide in 607 samples collected in areas with different histories of mefloquine use from residents and from travelers returning to France. Number of *Pvmdr*-*1* copies correlated with drug use history. Treatment against *P. falciparum* exerts substantial collateral pressure against sympatric *P. vivax*, jeopardizing future use of mefloquine against *P. vivax*. A drug policy is needed that takes into consideration all co-endemic species of malaria parasites.

## Abstract 2

Use of mefloquine against *P. falciparum* jeopardizes its future use against *P. vivax*.

Since World War II, antimalarial drugs have been intensively used to prevent or treat malaria (*1*). As observed with other antimicrobial agents, their use (or frequent misuse, when malaria diagnosis was based only on clinical symptoms without parasitologic confirmation) led to the emergence, selection, and spread of resistant parasites (*2*). This resistance became a global problem during the 1960s, when *Plasmodium falciparum* parasites developed resistance to chloroquine, the most widely used antimalarial drug at that time (*3*). In particular, resistant parasites that emerged in the Greater Mekong subregion of Asia later spread to Africa, triggering a dramatic increase in malaria and malaria-related deaths, particularly among children (*4*). During the 1980s, a similar scenario was observed with sulfadoxine-pyrimethamine (SP) when this association was recommended to replace chloroquine as first-line treatment in uncomplicated falciparum malaria (*5*,*6*). Since then, biological and molecular investigations using laboratory and field isolates have demonstrated that resistance of *P. falciparum* to antimalarial drugs is mediated by 2 major mechanisms: 1) a modification of the parasite target (i.e., mutations in *dihydrofolate reductase* [*dhfr*] or in *dihydropteroate synthetase* [*dhps*] genes) or 2) an increase of the efflux of the drug away from its site of action (i.e., mutations in the *chloroquine resistant transporter* gene or the *multidrug resistance-1* [*mdr-1*] gene or in an increased number of copies of the *mdr-1* gene). These molecular events have been intensively studied and are well known for *P. falciparum* but not for other *Plasmodium* species, mainly because of the ability to culture in vitro *P. falciparum* erythrocytic stages.

Our understanding of the molecular mechanisms of antimalarial drug resistance developed by *P. vivax* is less comprehensive. Although *Aotus* and *Saimiri* monkey models have provided useful information about *P. vivax* biology, most of the data have been gained through comparative studies investigating polymorphisms in orthologous genes encoding resistance to pyrimethamine (*dhfr* gene), sulfadoxine (*dhps* gene), or chloroquine (*chloroquine resistant transporter* or *mdr-1* genes). For instance, mutations in codons 57, 58, 61, 117, and 173 of *P. vivax* DHFR (corresponding to codons 51, 59, 108, and 164 of *P. falciparum* DHFR) are involved in resistance to pyrimethamine, although *P. vivax* infections are not usually treated directly with SP (*7*). This resistance was confirmed by heterologous expression studies, invalidating the common idea that *P. vivax* was “intrinsically resistant” to pyrimethamine (*8*), which suggests that the high frequency of mixed *P. falciparum*/*P. vivax* infections that are not detected by microscopy (*9*–*11*) or relapses of *P. vivax* infection after *P. falciparum* infections probably exposes *P. vivax* parasites to antimalarial drugs used to treat falciparum malaria infections, especially those with a long half-life, and selects *P. vivax* genetic traits conferring antimalarial drug resistance.

The impact of antimalarial drugs, especially those with long half-lives (such as mefloquine), on the sympatric *Plasmodium* species is not clearly understood. In areas where *P. falciparum* and *P. vivax* are co-endemic, such as South America and Southeast Asia, mefloquine has been widely used (alone in monotherapy or in combination with artemisinin derivatives) to treat uncomplicated falciparum malaria (*12*). In both areas, emergence of *P. falciparum* parasites resistant to mefloquine has been demonstrated from therapeutic efficacy studies (treatment failure) or in vitro testing (increased IC_50_ [half maximal inhibitory concentration]) and has been associated with the amplification of *P. falciparum mdr-1* (*Pfmdr-1*) gene (*13*–*16*). Recently, several studies performed on *P. vivax* samples collected in Southeast Asia (Thailand, Laos, Cambodia, and Myanmar) (*17*–*19*), South America (Brazil, Honduras) (*20*,*21*), and Africa (Mauritania) (*22*) have shown that *mdr-1* amplification does occur in *P. vivax*.

In this context, and to confirm the impact of the mefloquine drug pressure on *P. vivax* parasite populations, we used a real-time PCR to assess the number of *P. vivax mdr-1* (*Pvmdr-1*) gene copies to evaluate the worldwide distribution of *Pvmdr-1* amplification in samples collected from travelers with vivax malaria returning to France and from residents in areas with different histories of mefloquine use (French Guiana, Cambodia, Madagascar, and Sudan).

## Materials and Methods

### Sample Collection

*P. vivax* and *P. falciparum* samples from Madagascar were collected during 2006–2007 as part of the antimalarial drug resistance network, from symptomatic patients before treatment in 19 health centers located in areas of Madagascar with different epidemiologic patterns of malaria transmission: northern (Antsiranana, Antsohihy, Andapa), western (Mahajunga, Miandrivazo, Maevatanana, Morondava, Tsiroanomandidy, Ampasimpotsy), central (Saharevo, Moramanga), southern (Ihosy, Ejeda, Tolagnaro, Iakora, Ranostara, Toliara), and eastern (Farafangana, Toamasina). In Cambodia, *P. vivax* and *P. falciparum* isolates were obtained from symptomatic persons during 2010 in Pailin and Kratie Provinces. Other *P. vivax* samples were collected 1) from malaria-infected travelers returning to France after visiting Africa (Côte d’Ivoire, Ethiopia, Madagascar, and Mauritania), South America (Bolivia, Brazil, Colombia, French Guiana, Venezuela), and Asia (Bangladesh, Cambodia, India, Indonesia, Laos, Malaysia, Nepal, Pakistan, Sri Lanka) during 1997–2009 and were provided by the National Reference Center for Malaria (Paris, France); and 2) from symptomatic *P. vivax*–infected persons in French Guiana (2000–2003) or Sudan (2007).

### DNA Extraction and PCR Detection of *P. falciparum* and *P. vivax*

We extracted parasite DNA from blood spots with Instagene Matrix (BioRad, Marnes-la-Coquette, France) or from whole blood samples using the phenol-chloroform method (*23*) or the QIAamp DNA Blood Mini Kit (QIAGEN, Courtaboeuf, France), according to the manufacturer’s instructions. Molecular detection and identification of *Plasmodium* parasites were performed by using real-time PCR as described by Chou et al. (*24*).

### Determination of the Number of *Pfmdr-1* Copies in Isolates from Cambodia and Malagasy

We measured number of *Pfmdr-1* copies using CFX96 real-time PCR (BioRad) relative to the single copy of the *β-tubulin* (used as a reference gene). Briefly, PCRs were conducted in 25-μL volumes in a 96-well plate containing 1X HOT FIREPol EvaGreen qPCR Mix Plus (Solis BioDyne, Tartu, Estonia), 0.3 μM of each forward and reverse primer (*Pfmdr-1*, 5′-TGCATCTATAAAACGATCAGACAAA-3′ and 5′-TCGTGTGTTCCATGTGACTGT-3′; *β-tubulin*, 5′-TGATGTGCGCAAGTGATCC-3′ and 5′-TCCTTTGTGGACATTCTTCCTC-3′), and 4 μL of template DNA. Amplifications were performed under the following conditions: 94°C for 15 min, followed by 40 cycles of 94°C for 15 s, 58°C for 20 s, and 72°C for 20 s. The number of *Pfmdr-1* copies of each sample was measured in triplicate relative to a standard curve by using 4 standards of mixed plasmids cloned into TOPO cloning vector (Invitrogen, Saint Aubin, France): standard 1 (1:1 ratio of *Pfmdr-1* and β-tubulin), standard 2 (2:1 ratio of *Pfmdr-1* and *β-tubulin*), standard 3 (3:1 ratio of *Pfmdr-1* and *β-tubulin*) and standard 4 (4:1 ratio of *Pfmdr-1* and *β-tubulin*) and 2 parasite clonal lines used as controls, the 3D7 Africa line (1 copy of *Pfmdr-1*) and line Dd2 (3 copies of *Pfmdr-1*), by the ∆CT method (where CT is the cycle threshold). We defined >1.6 copies as a duplication of the gene.

### Evaluation of the Number of *Pvmdr-1* Copies

**Figure 1:**
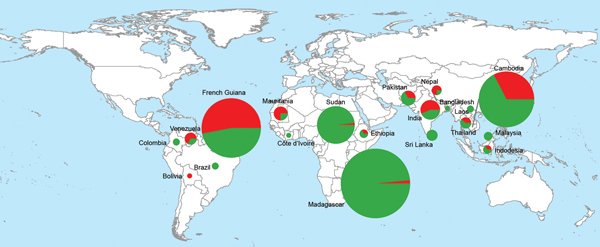
Geographic distribution of *Plasmodium vivax* isolates with 1 multidrug resistance-1 (*mdr-1*) copy (green) and isolates with >1 *mdr-1* copies (red) in 607 samples collected in South America, Asia, and Africa during 1997–2010.

We measured number of *Pvmdr-1* (*Pfmdr-1*) copies following the same procedure, relative to the single copy of the *β-tubulin*. Briefly, PCRs were conducted in 25-μL volumes in a 96-well plate containing 1X HOT FIREPol EvaGreen qPCR Mix Plus (Solis BioDyne), 0.3 μM of each forward and reverse primer (*Pvmdr-1*, 5′-GCAACTCCATAAAGAACAACATCA-3′ and 5′-TTTGAGAAGAAAAACCATCTTCG-3′; *β-tubulin*, 5′-CATGTTCGTTAAGATTTCCTGGT-3′ and 5′-GTTAGTGGTGCAAAACCAATCA-3′), and 4 μL of template DNA. Amplifications were performed under the following conditions: 94°C for 15 min, followed by 45 cycles of 94°C for 15 s, 59°C for 30 s, and 72°C for 30 s. The number of *Pvmdr-1* copies of each sample was measured in triplicate relative to a standard curve by using 6 standards of mixed plasmids cloned into TOPO cloning as described for *Pfmdr*-1 (from the standard-1, 1:1 ratio of *Pvmdr-1* and *β-tubulin* to the standard-6, 6:1 ratio of *Pvmdr-1* and *β-tubulin*) by the ∆CT method (Technical Appendix, Figure 1). We defined >1.6 copies as a duplication of the gene.

### Statistical Analysis

Microsoft Excel 2010 (Microsoft, Redmond, WA, USA) and MedCalc software (v9.1.0.1, Mariakerke, Belgium) were used for data analysis. Categorical variables were compared by χ^2^ test, and continuous variables were compared by using the 1-way analysis of variance or Mann-Whitney U test. We considered p values <0.05 as significant.

### Ethical Approval

We obtained ethics clearance for the samples used in this study from National Ethics Committee in Cambodia (Ministry of Health), in Madagascar (Ministry of Health), in Sudan (Ministry of Health), and in France (National Reference Center for Malaria). All patients or their parents/guardians provided informed written consent.

## Results

### Global Distribution of the Number of *Pvmdr-1* Copies

We collected and analyzed 607 *P. vivax* isolates from areas to which the parasite was endemic from a total of 492 residents (117 in South America;, 117 in Asia; and 258 in Africa) and 115 travelers from France to South America (41 travelers), Asia (60), or Africa (14). The number of *Pvmdr-1* copies ranged from 1 to 5 copies (mean 1.28, 95% CI 1.22–1.34; median 1.05 [interquartile range (IQR) 0.84–1.53]) and was distributed as follows: 75% isolates had 1 copy, 18% had 2 copies, 6% had 3 copies, 1.6% had 4 copies, and 0.4% had 5 copies. The frequency of *Pvmdr-1*–amplified isolates was significantly higher in samples from South America (83 [53%] of 158) than in samples from Asia (60 [34%] of 177, p = 10^−3^) or Africa (11 [4.0%] of 272, p<10^−5^). The mean number of *Pvmdr-1* copies was also higher in isolates from South America (1.8) than in isolates from Asia (1.3, p = 0.0007) or Africa (0.9, p<10^−5^). Number of copies differed significantly between *P. vivax* isolates from residents and those from travelers. In South America, the proportion of isolates with >1 copy of *Pvmdr-1* was significantly lower in travelers (34% vs. 59%, odds ratio [OR] 0.4 [95% CI 0.2–0.8], p = 0.007); in Africa, this proportion was significantly higher in travelers (57% vs. 1%, OR 113, 95% CI 24–536, p<0.0001) (Table 1, Table 2; Figure 1).

**Table 1:** Variation in number of copies of *Plasmodium vivax mdr-1* gene in 607 isolates from residents of and travelers from France to malaria-endemic areas, South America, Asia, and Africa, 1997–2009

Variable	Residents	Travelers	Total	p value
No. isolates	492	115	607	–
Origin of isolate, no. (%)				
South America	117 (24)	41 (36)	158 (26)	–
Asia	117 (24)	60 (52)	177 (29)	
Africa	258 (52)	14 (12)	272 (45)	
Isolates with >1 *Pvmdr-1* copy				
No. (%; 95% CI)	109 (22; 18–26)	45 (39; 30–49)	154 (25; 22–29)	<10^–3^*
Range	1–5	1–3	1–5	
Copies of *Pvmdr-1*, no. (%)				
1	383 (77.9)	70 (61)	453 (75)	<10^–8^†
2	63 (12.8)	44 (38)	108 (18)	
3	34 (6.9)	1 (1)	35 (6)	
4	10 (2.0)	0	10 (1.6)	
5	2 (0.4)	0	2 (0.4)	
Mean (95% CI)	1.27 (1.21–1.34)	1.32 (1.22–1.41)	1.28 (1.22–1.34)	0.57‡

*χ^2^ test. †1-way analysis of variance. ‡Mann-Whitney U test.

**Table 2:** Variation in number of copies of *Plasmodium vivax mdr-1* gene among isolates from residents of malaria-endemic continents and from travelers from France to those areas, 1997–2010

Continent	Residents	Travelers	Total	p value
South America				
Isolates with >1 *Pvmdr-1* copy	117	41	158	
No. (%; 95% CI)	69 (59; 50–68)	14 (34; 20–51)	83 (53; 44–60)	0.01*
Range	1–5	1–2	1–5	
Copies, no. (%)				
1	48 (41)	27 (66)	75 (47)	10^–4^*
2	26 (22)	14 (34)	40 (25)	
3	32 (27)	0	32 (20)	
4	9 (8)	0	9 (6)	
5	2 (2)	0	2 (1)	
Mean (95% CI)	2.0 (1.8–2.2)	1.2 (1.0–1.3)	1.8 (1.7–2.0)	<10^–5^†
Asia				
Isolates with >1 *Pvmdr-1* copy	117	60	177	
No. (%; 95% CI)	37 (32; 23–45)	23 (38; 24–57)	60 (34; 26–44)	0.5*
Range	1–4	1–3	1–4	
Copies, no. (%)				
1	80 (68)	37 (62)	117 (66)	0.68*
2	34 (29)	22 (37)	56 (32)	
3	2 (2)	1 (2)	3 (2)	
4	1 (1)	0	1 (1)	
Mean (95% CI)	1.3 (1.2–1.4)	1.4 (1.3–1.5)	1.3 (1.2–1.4)	0.41†
Africa				
Isolates with >1 *Pvmdr-1* copy	258	14	272	
No. (%; 95% CI)	3 (1; 0.2–3)	8 (57; 24–100)	11 (4; 2–7)	<10^–8^*
Range	1–2	1–2	1–2	
Copies, no. (%)				
1	255 (99)	6 (43)	261 (96)	<10^–8^*
2	3 (1)	8 (57)	11 (4)	
Mean (95% CI)	0.9 (0.9–1.0)	1.3 (1.1–1.6)	0.9 (0.9–1.0)	<10^–8^†

*χ^2^ test. †1-Way analysis of variance.

### Amplification of *Pvmdr-1* in Isolates from Residents

Number of copies in isolates from residents differed significantly among continents (Table 3). The mean number of *Pvmdr-1* copies was highest in isolates from South America (2.04, p<10^−5^ vs. Asia and Africa), followed by Asia (1.32, p<10^−4^ vs. Africa) and Africa (0.90 for Madagascar and 1.0 for Sudan). In South America, the proportion of isolates with >1 *Pvmdr-1* copies was also more frequent (59%) than in Asia (33%, OR 0.33, 95% CI 0.2–0.6, p = 10^−4^) and Africa (1%, OR 0.008, 95% CI 0.002–0.02, p<10^−5^ vs. South America; OR = 0.02, 95% CI 0.007–0.08, p<10^−4^ vs. Asia).

**Table 3:** Variation in number of copies of *Plasmodium vivax mdr-1* gene in isolates from residents of selected Asian and African countries

Variable	Country, years of sample collection				Total	p value
	French Guiana, 2000–2003	Cambodia, 2010	Madagascar, 2006–2007	Sudan, 2007		
No. isolates	117	117	199	59	492	
Isolates with >1 *Pvmdr-1* copy						
No. (%; 95% CI)	69 (59; 46–75)	37 (32; 23–45)	2 (1; 0.1–3.6)	1 (2; 0.4–9.5)	109 (22; 18–26)	<10^–10^*
Range	1–5	1–4	1–2	1–2	1–5	
Copies*,* no. (%)						<10^–10^*
1	48 (41)	80 (67)	198 (99)	57 (98)	383 (77.9)	
2	26 (22)	34 (30)	1 (1)	2 (2)	63 (12.8)	
3	32 (27)	2 (2)	0	0	34 (6.9)	
4	9 (7)	1 (1)	0	0	10 (2.0)	
5	2 (3)	0	0	0	2 (0.4)	
Mean (95% CI)	2.04 (1.85–2.24)	1.32 (1.22–1.43)	0.90 (0.84–0.91)	1.00 (0.94–1.05)	1.27 (1.21–1.34)	<10^–10^†

*χ^2^ test. †1-Way analysis of variance.

### Amplification of *Pvmdr-1* in Isolates from Travelers

The mean number of *Pvmdr-1* copies was similar for travelers returning to France from South America or Asia and did not change by year of collection: 1997–2000 (1.42 and 1.24, respectively), 2001–2005 (1.17 and 1.38), and 2006–2009 (1.20 and 1.49). In contrast, for travelers returning to France from Africa, the mean number of *Pvmdr-1* copies increased in samples collected more recently: 0.60 during 1997–2000, 1.02 during 2001–2005, and 1.48 during 2006–2009 (OR 0.15, 95% CI 0.03–0.71, p = 0.049). The proportion of isolates with >1 copies of *Pvmdr-1* did not differ significantly among continents over time (Table 4).

**Table 4:** Variation in number of copies of *Plasmodium vivax mdr-1* gene among isolates from persons from France who reported having traveled during the previous month to malaria-endemic continents, 1997–2010

Variable	South America	Asia	Africa	Total	p value
No. isolates	41	60	14	115	
Isolates with >1 *Pvmdr-1* copy					
No. (%; 95% CI)	14 (34; 18–57)	23 (38; 24–57)	8 (57; 24–100)	45 (39; 30–49)	0.30*
Range	1–2	1–3	1–2	1–3	
Copies, no. (%)					0.49*
1	26 (63)	37 (62)	6 (43)	70 (61)	
2	14 (34)	22 (37)	8 (57)	44 (38)	
3	0	1 (2)	0	1 (1)	
Mean (95% CI)	1.20 (1.05–1.35)	1.40 (1.27–1.53)	1.35 (1.10–1.59)	1.32 (1.22–1.41)	0.12†

*χ^2^ test. †1-Way analysis of variance.

### Amplification of *Pvmdr-1* and *Pfmdr-1* in Isolates from Cambodia and Malagasy

**Figure 2:**
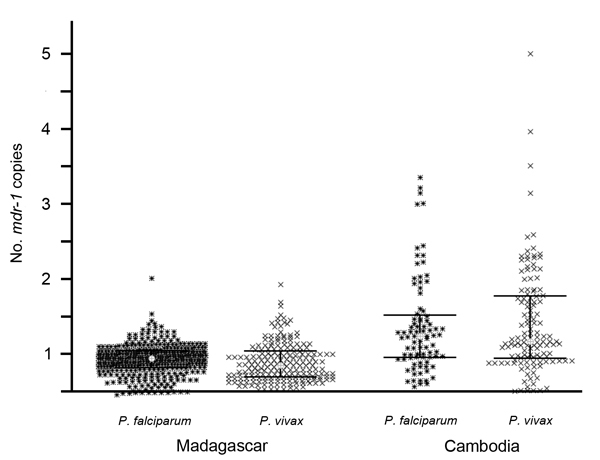
Comparative distribution of numbers of *Plasmodium falciparum* and *P. vivax mdr-1* copies in isolates collected from residents of Madagascar and Cambodia. Gray dot, median; dark bars, interquartile range (25%–75%).

We compared the distribution profile of the number of *mdr-1* copies of *P. falciparum* and *P. vivax* isolates in 2 different settings. In both Cambodia and Madagascar, the number of *mdr-1* copies did not differ between the 2 species (Figure 2). In Cambodia, where mefloquine has been widely used for >25 years, the mean of number of *mdr-1* copies was 1.34 for *P. falciparum* (n = 88, 95% CI 1.22–1.47, range 1–3) and 1.34 for *P. vivax* (n = 129, 95% CI 1.24–1.44, range 1–4, p = 0.52). In contrast, in Madagascar where mefloquine has never been recommended and has been barely used, the mean number of *mdr-1* copies was 0.92 for *P. falciparum* (n = 350, 95% CI 0.90–0.94, range 1–2) and 0.90 for *P. vivax* (n = 201, 95% CI 0.87–0.93, range 1–2, p = 0.21).

## Discussion

Developed in the 1970s at the US Department of Defense’s Walter Reed Army Institute of Research as a synthetic analog of quinine (*25*), mefloquine was introduced in 1983 in Thailand to replace chloroquine as first-line treatment for falciparum malaria (*26*). Since then, mefloquine alone or in combination with artesunate has been widely used, especially in Southeast Asia (including Cambodia) and South America (including French Guiana), where it was introduced for second-line treatment and for chemoprophylaxis in 1990 (*27*). In contrast, mefloquine has not been used extensively in Africa and has not been introduced in Madagascar. Mefloquine has been available for malaria chemoprophylaxis since 1985 in Europe and since 1990 in the United States and has been used by >35 million travelers from France for this indication (*28*,*29*).

*Pfmdr-1* gene amplification has been described as the major mechanism of *P. falciparum* mefloquine resistance associated with treatment failure or in vitro resistance (*13*–*16*). Previous studies, including ours, confirm that *mdr-1* amplification does occur in *P. vivax (**17**–**22**)*. In addition, the epidemiologic data in our current study show that in regions where mefloquine has never been used, such as in Madagascar and Sudan, amplification in *Pvmdr*-1 is rare (1% and 2% of total isolates, respectively), whereas in areas with current or past intense use of mefloquine, such as in French Guiana and Cambodia, *Pvmdr*-1 amplification is frequent and detected in 59% and 33% of isolates, respectively, and with a mean of 2 and 1.3 copies, respectively.

Both the number of copies and prevalence of *Pvmdr*-1 of isolates with multiple *mdr*-1 copies we observed here are much higher than reported in other studies. For instance, Imwong et al. reported *Pvmdr-1* amplification in 6/66, 2/50, and 1/49 isolates from Thailand, Laos, and Myanmar, respectively (*17*); Jovel et al. observed *Pvmdr-1* amplification in 1/37 in Honduras (*20*); and Lin et al. recently reported 39% and only 4% prevalence in *P. vivax* isolates from Thailand and Cambodia, respectively (*18*). A reason for the discrepancy between observations from Lin et al. in Cambodia and our observations could be because their isolates were collected during 2006–2007 and our samples were collected in 2010, indicating an increase in *Pvmdr-1* amplification over 3 years. Another reason is the location of collection. Indeed, drug resistance and drug pressure markedly differ across Cambodia. Lin et al. studied isolates from southern Cambodia (Kampot Province), whereas we studied isolates from areas in which drugs were highly resistant in western (Pailin Province) and southeastern Cambodia (Kratie Province), where multidrug resistance of *P falciparum* is emerging (*30*) and drug pressure (including artesunate–mefloquine combination) has been intense in recent years. To our knowledge, the previous maximum number of *Pvmdr-1* copies detected was 3 (*18*,*19*); in this study. however, we observed up to 5 copies in isolates from South America and 4 copies in isolates from Southeast Asia. This difference is likely to be due to our real-time PCR approach using 6 standards of mixed plasmids, which enabled detection of *P. vivax* isolates with *mdr-1* amplification with up to 6 copies. This observation also could indicate an ongoing selection of *mdr-1*–amplified parasites. Although, our data need to be confirmed and supported by in vivo data from mefloquine-treated patients or in vitro experiments showing a direct relationship between mefloquine pressure and *P. vivax* *mdr-1* amplification, our findings advocate for an integrated drug policy whereby all sympatric malaria species are considered regarding treatment efficacy but also drug pressure and selection of resistance.

We were able to assess the number of *Pvmdr-1* copies in isolates collected from travelers returning to France. These data must be analyzed with caution because information about the location of infection might be erroneous, particularly given the fact that relapses from hypnozoites can occur several months after primary infection. We cannot exclude that a patient declaring having returned from Africa was previously infected during a trip to a different location because we did not include this information in the questionnaire administered at recruitment. Nevertheless, we found significant differences between travelers from France and residents from a given geographic origin, especially in isolates from Africa, where most (99%) isolates from residents displayed no amplification, whereas most (57%) isolates from travelers from France had *Pvmdr-1* amplification. We assume this indicates within-host selection by mefloquine prophylaxis, which has been and continues to be widely used among travelers from France who go to malaria-endemic countries. Such pressure does not exist in residents, who usually do not take any prophylaxis. These data are of concern because they suggest that selection of *Pvmdr-1* amplification is a more rapid process than previously thought, reminiscent to atovaquone resistance. Although selection in travelers from France returning to nonendemic areas bears no transmission risk, chemoprophylaxis and intermittent preventive treatments in malaria-endemic areas might contribute to the emergence of resistant parasites. This possibility certainly warrants further investigation.

Technical Appendix Evaluation of the number of *Plasmodium vivax mdr-1* copies by using a standard curve of 6 standards of mixed plasmids (from the standard-1, 1:1 ratio of *Pvmdr-1* and *β-tubulin* to the standard-6, 6:1 ratio of *Pvmdr-1* and *β-tubulin*) by the ΔCT method.
