# Supplementary material for: Effects of Mefloquine Use on Plasmodium vivax Multidrug Resistance
Source: Emerg Infect Dis. 2014 Oct;20(10):1637–44. doi: 10.3201/eid2010.140411 (PMC4193276; doi:10.3201/eid2010.140411)
Supplement: Technical Appendix — Evaluation of the number of Plasmodium vivax mdr-1 copies by using a standard curve of 6 standards of mixed plasmids (from the standard-1, 1:1 ratio of Pvmdr-1 and β-tubulin to the standard-6, 6:1 ratio of Pvmdr-1 and β-tubulin) by the ΔCT method. [file 14-0411-Techapp-s1.pdf]

Article DOI: http://dx.doi.org/10.3201/eid2010.140411

Nontargeted Effects of Mefloquine Use on *Plasmodium vivax* Multidrug Resistance

Technical Appendix

Standards	Standard Pvmdr-1 copy number						sample 1	sample 2
	1	2	3	4	5	6		
$CT_{Pvmdr-1}$	28.2 28.3 28.2	25.7 25.1 25.4	25.1 24.9 25.1	24.0 24.0 24.0	25.8 26.0 25.8	24.6 24.0 24.9	34.9 35.4 35.1	21.0 20.7 21.2
Mean $CT_{Pvmdr-1}$	28.2	25.4	25.0	24.0	25.9	24.5	35.1	21,0
$CT_{\beta\text{-tubulin}}$	26.2 26.0 25.7	26.3 26.1 26.0	26.6 26.0 26.8	25.9 26.1 25.9	27.8 28.2 27.96	27.3 26.8 26.8	31.4 31.5 31.5	22.7 23.1 22.8
Mean $CT_{\beta\text{-tubulin}}$	25.9	26.1	26.5	25.9	28.0	26.9	31.5	22.9
ΔCT ($CT_{Pvmdr-1}$-$CT_{\beta\text{-tubulin}}$)	2.3	-0.75	2.7	-1,9	-2.1	-2.4	3.6	-1.9
$2^{-\Delta CT}$	0.2	1.7	2.7	3.9	4.3	5.5	0.1	3.7
Estimated Pvmdr-1 copy number	0.8	2.1	3.2	4.3	4.9	5.9	0.7	4.1

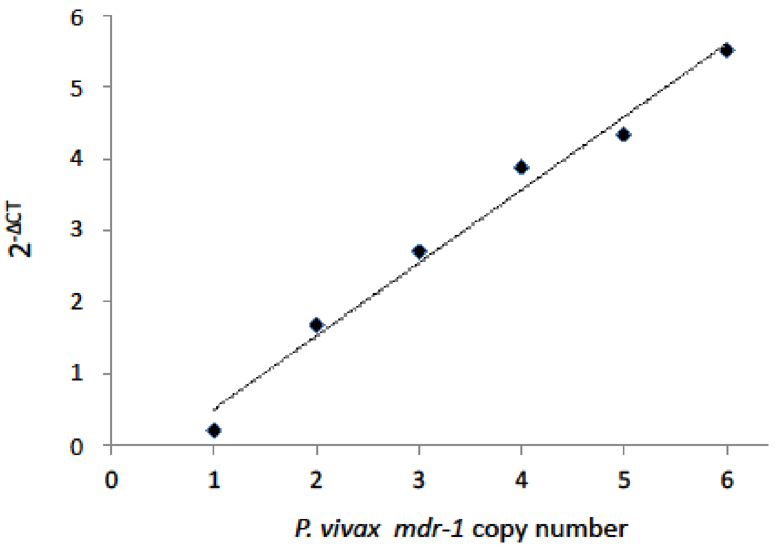

Technical Appendix Figure. Evaluation of the number of *Plasmodium vivax mdr-1* copies by using a standard curve of 6 standards of mixed plasmids (from the standard-1, 1:1 ratio of *Pvmdr-1* and *β-tubulin* to the standard-6, 6:1 ratio of *Pvmdr-1* and *β-tubulin*) by the ΔCT method.
